# The association between social media for medical information during pregnancy on maternal mental health at the end of the third trimester

**DOI:** 10.1007/s00737-025-01608-8

**Published:** 2025-07-05

**Authors:** Riley Huddleston, Maya Julian-Kwong, Marcelle I. Cedars, Eleni G. Jaswa, Maren Shapiro Eger, Anna Sindalovsky, Katherine Geisler, Heather G. Huddleston, Jamie Corley, Elena Hoskin, Karla J. Lindquist

**Affiliations:** 1 Depertment of Integrative Biology, University of Wisconsin, Madison, WI USA; 2 Department of Neuroscience, Brown University, Providence, RI USA; 3 Center for Reproductive Health, University of California, San Francisco, CA USA; 4 School of Public Health, University of California, Berkeley, CA USA

**Keywords:** Social media use, Prenatal anxiety, Prenatal distress, Prenatal depression

## Abstract

This study assessed the correlation between social media use for medical information during pregnancy and maternal mental health. We found that prenatal social media use for medical information was associated with higher anxiety and distress symptoms at the end of pregnancy. Healthcare professionals should be prepared to counsel women on using social media for medical information during pregnancy.

## Introduction

Pregnancy is a uniquely high-risk period for mental health. Antenatal depression has a prevalence of approximately 7–20% in high-income countries such as the United States (Biaggi et al. 2016). Antenatal distress, defined as maternal psychological adversities, including stress, anxiety, and depression, is linked to lower birth weights, reduced gestational age, and increased miscarriage risk (Khoury et al. 2022). Additionally, some studies suggest antenatal distress and infant neurodevelopmental associations (Wu et al. 2020). Risk factors for poor mental health during pregnancy include lack of social support, history of mental illness, high-stress events, unwanted or unplanned pregnancy, pregnancy complications, and domestic abuse (Biaggi et al. 2016).

The widespread uptake of social media represents a profound shift in how individuals gather information. The COVID-19 pandemic heightened the use of social media, including for medical information, due to its accessibility during times of isolation (Caddy et al. 2023). Growing concern surrounds the potential effects of social media use (SMU), as it is increasingly linked to issues such as lowered self-esteem, increased risk of addiction, depression, and other psychological disorders (Zsila and Reyes 2023). Pregnancy is an experience that increases information-seeking behavior. However, the degree to which SMU for medical information impacts one’s psychological well-being during pregnancy is unclear. Most studies addressing the impact of SMU have not explicitly addressed the consumption of medical information. Only one cross-sectional study in China during the pandemic found that use of a hospital social media site for antenatal health information was associated with a lower risk of mental health challenges (Jiang et al. 2021).

In short, SMU for medical information’s rise over the past decade has outpaced our understanding of benefits and risks. Given the vulnerability of pregnant people to mental health challenges and the potential downstream implications, understanding emerging factors that could contribute to adverse mental health is critical. Accordingly, this prospective study aims to assess the correlation between SMU for medical information during pregnancy and mental health at the end of pregnancy. Based on emerging links between social media and mental health, we hypothesized that SMU for medical information would be correlated with worsened mental health outcomes.

## Materials and methods

### Study design and participants

The ASPIRE study is a prospective cohort study (April 2020–August 2021) recruiting participants through clinical and web-based advertisements. Eligible participants were *≥* 18 years, < 10 weeks pregnant, and provided written consent. Surveys were conducted at enrollment and at the end of each trimester, assessing health and behaviors during the COVID-19 pandemic. This analysis included those who completed baseline demographics, mental health surveys (baseline, third trimester), and a questionnaire on medical information sources. Surveys were administered electronically via REDCaP. The study was approved by the UCSF IRB (#20-30559).

### Mental health outcomes

Third-trimester mental health was assessed using the General Anxiety Disorder-7 (GAD-7) (Spitzer et al., 2006), Revised Prenatal Distress Questionnaire (NuPDQ-17) (Lobel et al. 2008), and Patient Health Questionnaire-9 (PHQ-9) (Kroenke et al. 2001), with higher scores indicating more significant anxiety (0–21), distress (0–34), and depression (0–27). All have reported Cronbach’s alpha reliabilities of > 0.8 (Johnson et al. 2019; Ibrahim and Lobel 2019; Kroenke et al. 2001; respectively). Questionnaires were self-administered online.

### Main exposure and covariates

Our primary exposure of interest was SMU *for medical information*. Our exposure was determined by a third-trimester questionnaire that asked participants: “During your pregnancy, which of the following sources did you use to obtain medical information?” Sources included social media (e.g., blogs, Facebook, Instagram, YouTube, and others), healthcare providers, friends/family, and other websites. Covariates were selected a priori and included recruitment source, maternal age, race/ethnicity, education, income, children at home, healthcare employment, baseline mental health scores, and frequency of internet use for medical information (a 5-point Likert scale ranging from never to very frequently).

### Statistical analysis

Bivariable associations between SMU for medical information and baseline characteristics were compared using linear and logistic regression for continuous and categorical characteristics, respectively, adjusting for other sources of medical information (other websites, health care providers, and friends and family). Linear regression models tested associations between SMU and each mental health outcome, adjusting for other medical information sources (Model 1), internet use for medical information frequency (Model 2), and demographics (Model 3), with bootstrapped standard errors (1,000 repetitions). Effect modification by frequency of internet use was tested using an interaction term between the indicator for SMU for medical information and the 5-level frequency of use variable in the adjusted models. A supplementary analysis was performed to test the association between mental health and SMU as the *primary source* of medical information during pregnancy, adjusting for the same baseline characteristics used in the other models.

Analyses were conducted in R 4.3.2 and Stata/BE 18.0. Significance was assessed with *p* < 0.05 and 95% confidence intervals.

## Results

2,749 ASPIRE participants qualified for analysis, with 52.5% (*N* = 1,442) using social media for medical information. The latter were more likely to be Hispanic (*p* = 0.004), without children at home (*p* = 0.019), not working in healthcare (*p* < 0.001), and reported more frequent internet use for medical information (trend *p* < 0.001). Participants in the SMU for medical information group also had higher baseline GAD-7, NuPDQ-17, and PHQ-9 scores (Table 1) than those who did not use social media for medical information. While most (94.0%) used social media during pregnancy, only 4.6% relied on it as their primary source of medical information.

**Table 1:** Baseline characteristics by social media use for medical information during pregnancy

Characteristic or Group	Overall Cohort (*N* = 2,749)	No Social Media Use (*N* = 1,307)	Social Media Use (*N* = 1,442)	*P*-value*
	Mean (SD) or N (%)	Mean (SD) or *N* (Row %)		
**Sample recruitment source**				
Community (BabyCenter)	2046 (74.4)	972 (47.5)	1074 (52.5)	0.611
SART	700 (25.5)	334 (47.7)	366 (52.3)	
**Maternal age (years)**	33.3 (4.2)	33.5 (4.2)	33.1 (4.2)	0.063
**Maternal race**				0.831
Asian	117 (4.3)	55 (47.0)	62 (53.0)	
Black	58 (2.1)	30 (51.7)	28 (48.3)	
White	2418 (88.0)	1151 (47.6)	1267 (52.4)	
Multiethnic/Other	103 (3.8)	45 (43.7)	58 (56.3)	
**Maternal ethnicity**				
Not Hispanic	2449 (89.1)	1180 (48.2)	1269 (51.8)	0.004
Hispanic	233 (8.5)	91 (39.1)	142 (60.9)	
**Maternal education**				
Less than Bachelor’s degree	354 (12.9)	177 (50.0)	177 (50.0)	0.438
Bachelor’s degree	922 (33.5)	422 (45.8)	500 (54.2)	
Graduate degree	1459 (53.1)	700 (48.0)	759 (52.0)	
**Household income**				
<$50,000	220 (8.0)	106 (48.2)	114 (51.8)	0.908
$50,000-$99,000	687 (25.0)	328 (47.8)	359 (52.3)	
$100,000-$250,000	1432 (52.1)	686 (47.9)	746 (52.1)	
>$250,000	397 (14.4)	180 (45.3)	217 (54.7)	
**Has other children at home**				
No	874 (31.8)	378 (43.2)	497 (56.8)	0.019
Yes	1869 (68.0)	926 (49.7)	942 (50.4)	
**Works in healthcare field (patient care role)**				
No	1410 (51.3)	602 (42.7)	808 (57.3)	< 0.001
Yes	539 (19.6)	308 (57.1)	231 (42.9)	
**Frequency of internet use for medical information**				
Never	549 (20.0)	439 (80.0)	110 (20.0)	
Rarely	892 (32.5)	441 (49.4)	451 (50.6)	< 0.001
Occasionally	833 (30.3)	293 (35.2)	540 (64.8)	
Somewhat frequently	359 (13.1)	97 (27.0)	262 (73.0)	
Very frequently	116 (4.2)	37 (31.9)	79 (68.1)	
**General anxiety/worry level (GAD-7)**				
Raw score (0–21)	4.9 (3.9)	3.7 (3.8)	4.3 (4.0)	0.001
**Prenatal distress level (NuPDQ-17)**				
Raw score (0–34)	10.4 (6.2)	9.9 (6.0)	10.9 (6.3)	< 0.001
**Depression level (PHQ-9)**				
Raw score (0–27)	5.1 (3.7)	4.9 (3.7)	5.3 (3.7)	0.002

*P-value is for use of social media for medical information, adjusted for use of other sources. Bootstrapped linear regression was used for continuous characteristics, binary or multinomial logistic regression for categorical characteristics

When evaluating raw mental health scores at the end of the third trimester, we found that those who used social media for medical information during pregnancy had more anxiety, depressive symptoms, and more prenatal distress. Mean ± standard deviation GAD-7 scores were 3.96 ± 3.98 versus 3.41 ± 3.85, respectively (t-test *p* < 0.001). PHQ-9 scores were 4.11 ± 3.51 versus 3.83 ± 3.59, respectively (*p* = 0.037). NuPDQ-17 scores were 10.41 ± 5.43 versus 9.08 ± 5.48, respectively (*p* < 0.001).

After adjusting for other sources of medical information (healthcare providers, friends/family, and other websites), baseline mental health scores, frequency of internet use for medical information, and demographics, SMU for medical information was still associated with significantly increased anxiety (GAD-7) and prenatal distress (NuPDQ-17) (Table 2). No association was found with depression (PHQ-9) after adjustment.

**Table 2:** Regression coefficients for standard deviation differences in third trimester mental health scores by source of medical information, adjusted for baseline characteristics

Covariate	General Anxiety (GAD-7)	Prenatal Distress (NuPDQ-17)	Depression (PHQ-9)
	Coefficient (95% CI)	Coefficient (95% CI)	Coefficient (95% CI)
Source of medical information used			
Social media (ref: does not use)	0.085 (0.001, 0.170)**	0.102 (0.022, 0.181)**	0.016 (-0.067, 0.099)
Other websites (ref: does not use)	0.058 (-0.089, 0.205)*	0.174 (-0.016, 0.364)*	0.075 (-0.085, 0.235)
Health provider (ref: does not use)	0.014 (-0.120, 0.147)	-0.002 (-0.140, 0.137)	-0.002 (-0.156, 0.152)
Friends and family (ref: does not use)	0.072 (-0.009, 0.152)	0.069 (-0.013, 0.152)	0.031 (-0.052, 0.113)
Baseline score (standardized)	0.559 (0.501, 0.617)***	0.585 (0.54, 0.631)***	0.489 (0.434, 0.543)***
Frequency of internet use for medical information			
Rarely (ref: never)	-0.019 (-0.122, 0.084)	0.015 (-0.100, 0.129)	-0.019 (-0.128, 0.090)
Occasionally (ref: never)	-0.023 (-0.137, 0.090)	0.026 (-0.087, 0.140)	-0.024 (-0.140, 0.093)
Somewhat frequently (ref: never)	0.082 (-0.060, 0.225)	0.065 (-0.083, 0.214)	0.082 (-0.060, 0.223)
Very frequently (ref: never)	0.190 (-0.081, 0.461)	0.190 (-0.027, 0.407)	0.189 (-0.092, 0.471)
Recruitment sample SART (ref: Community)	-0.090 (-0.175, -0.005)**	0.104 (0.008, 0.199)**	-0.004 (-0.093, 0.084)
Maternal age (years)	0.008 (-0.002, 0.018)	-0.002 (-0.012, 0.008)	0.002 (-0.008, 0.012)
Maternal race			
Asian (ref: White)	-0.060 (-0.228, 0.108)	0.277 (0.068, 0.487)***	-0.182 (-0.354, -0.009)**
Black (ref: White)	-0.342 (-0.564, -0.120)***	-0.248 (-0.530, 0.034)*	-0.315 (-0.591, -0.038)**
Multiethnic/other (ref: White)	0.093 (-0.159, 0.345)	0.142 (-0.111, 0.395)	0.015 (-0.222, 0.252)
Maternal ethnicity Hispanic (ref: non-Hispanic)	0.055 (-0.119, 0.230)	0.080 (-0.089, 0.249)	-0.014 (-0.194, 0.167)
Maternal education			
Bachelor’s degree (ref: < Bachelor’s)	-0.026 (-0.198, 0.146)	0.135 (-0.022, 0.291)*	-0.025 (-0.196, 0.146)
Graduate degree (ref: < Bachelor’s)	-0.017 (-0.188, 0.154)	0.212 (0.057, 0.368)***	-0.038 (-0.211, 0.135)
Household income			
$50-99k (ref: <$50k)	-0.136 (-0.370, 0.099)	-0.433 (-0.672, -0.193)***	-0.251 (-0.527, 0.026)*
$100-250k (ref: <$50k)	-0.203 (-0.432, 0.026)*	-0.480 (-0.718, -0.242)***	-0.263 (-0.535, 0.009)*
>$250k (ref: <$50k)	-0.267 (-0.513, -0.021)**	-0.441 (-0.691, -0.192)***	-0.334 (-0.618, -0.050)**
Has other children at home (ref: does not)	0.073 (-0.002, 0.148)*	0.148 (0.067, 0.228)***	0.128 (0.051, 0.206)***
Works in healthcare (ref: does not)	0.056 (-0.031, 0.143)	0.026 (-0.059, 0.111)	0.029 (-0.062, 0.119)

*p < 0.1, **p < 0.05, ***p < 0.01

CI: confidence interval. Positive coefficients correspond to higher (worse) mental health scores

GAD-7: General Anxiety Disorder-7

NuPDQ-17: Revised Prenatal Distress Questionnaire

PHQ-9: Patient Health Questionnaire-9

More frequent internet use for medical information on a 5-point Likert scale correlated with worse third-trimester mental health (Kruskal-Wallis *p* = 0.003 for GAD-7, *p* < 0.001 for PHQ-9, *p* < 0.001 for NuPDQ-17). However, no significant interaction was found between SMU and the frequency of internet use for medical information. Third-trimester mental health did not differ among those using social media as their primary medical source versus healthcare providers.

## Discussion

This study examined the relationship between SMU for medical information during pregnancy and mental health at the end of pregnancy. We found SMU for medical information during pregnancy was associated with worse general anxiety and antenatal distress symptoms in the third trimester. Those reporting SMU for medical information also had higher baseline (first trimester) mental health scores; however, after adjusting for this, SMU remained independently associated with adverse third-trimester mental health. These results suggest that SMU for medical information may exacerbate anxiety and distress in an already vulnerable group. Furthermore, other sources of medical information–healthcare providers, websites, friends and family–were not significantly associated with third-trimester mental health outcomes. This finding suggests the observed associations are specific to SMU rather than reassurance-seeking behavior.

Few studies have examined SMU for medical information during pregnancy. One retrospective study found that 89% of mothers reported SMU for parenting advice but not specifically for medical information (Baker and Yang 2018). Another reported that SMU in general increased among pregnant women following the pandemic, further establishing a need to understand its effects on mental health (Caddy et al. 2023). Another reported that higher general SMU was associated with prenatal depression (Muskens et al. 2023). Possible mechanisms proposed include exposure to inaccurate or overwhelming information.

The association between SMU for medical information and prenatal distress has significant implications. A review found pregnancy-specific stress was linked to obstetric complications, unplanned surgical delivery, and poor maternal and infant outcomes (Ibrahim and Lobel 2019). Given the impact of poor mental health on pregnancy outcomes, further research should explore how healthcare providers can mitigate the adverse effects of SMU, especially for seeking medical information. Clinicians should educate patients about reliable sources and contribute to evidence-based social media tools.

Our study’s strengths include the size and prospective design, focus on SMU specifically for medical information during pregnancy, and adjustments for confounders. One limitation is a lack of specificity on the type of medical information being sought through social media. Also, our reliance on self-reported measures may introduce misclassification bias. Additionally, our cohort skews toward a well-resourced population, limiting generalizability. Future studies could disentangle these relationships further in diverse settings. Conducted during the COVID-19 pandemic, our findings reflect a unique period of social isolation and heightened health concerns that may not generalize to current trends. Finally, given our observational design, causation cannot be determined, and the relationship between SMU and mental health is likely bidirectional. Future research should further explore these connections.

In summary, SMU for medical information during pregnancy is associated with higher third-trimester anxiety and distress, even after accounting for baseline mental health. This study underscores the importance of understanding SMU’s impact and exploring interventions to mitigate potential harms.
